# A. V. M. A.

**Published:** 1902-11

**Authors:** 


### A. V. M. A.

The clinics of the Minneapolis meeting awakened serious discussion along certain lines, and some doubts were aroused as to the value of certain operations.

The facilities for demonstration at the Experiment Station at St. Anthony Park were most excellent. Much of the value of these operations is lost by the difficulties attending the lack of knowledge of the after-progress and results of these operations.

Editor Bell, of the *American Veterinary Review*, was an interested spectator of the clinics. The need of more journalistic ammunition to sustain this feature of State or local work in the category of the National Association was the moving cause. Would it not be well for the A. V. M. A. to yearly appropriate a fund of money and send some one comparative surgeon into all the larger cities to give a post-graduate course to all the A. V. M. A. members?

There is a growing demand that in the future our meetings must be held in better arranged halls than are afforded by the hotels where our conventions have their headquarters.

The ride on Lake Minnetonka was a delightful experience, and there was some tripping of the light fantastic to the excellent music that recalled days gone by—times when less serious problems vexed those who now consider the problems of national veterinary sanitary control work.

President Winchester adds his large mite of testimony to the historic saying: "Uneasy lies the head that wears a crown." He was heard to say: "Oh! that I might be saved from being hunted by Wolverines."

All the way from Honolulu comes the announcement that the meeting of 1903 of the A. V. M. A. should be held in New Orleans, La. This looks like a pooling of issues of Members Monsarratt and Dalrymple.

The Dr. G. Ed. Leech poster announcement of the meeting at Minneapolis was in keeping with the enthusiastic nature of that association boomer. While an old Wisconsin graduate, his announcement was signed "One of the Minnesotas." Dr. Ellis, of St. Louis, has engaged Dr. Leech to inflate the boom for St. Louis in 1904.

Dr. J. G. Annand was most assiduous in his attentions to the ladies, and his many kindly courtesies were very highly appreciated.

Dr. W. W. Conkey, of Grand Rapids, Michigan, made a strong impression upon President Winchester, who became sponsor for the Association badge so proudly worn by Dr. Conkey.

